# The association of gut microbiota with body weight and body mass index in preschool children of Estonia

**DOI:** 10.3402/mehd.v24i0.19231

**Published:** 2013-02-20

**Authors:** Epp Sepp, Krista Lõivukene, Kaja Julge, Tiia Voor, Marika Mikelsaar

**Affiliations:** 1 Department of Microbiology, University of Tartu, Tartu, Estonia; 2 Children's Clinic of Tartu University Clinics, Tartu, Estonia

**Keywords:** BMI, weight, bifidobacteria, eubacteria, bacteroides, relative share of total cultivable intestinal microbiota

## Abstract

**Background:**

The gut microbiota has been shown to affect both fat storage and energy harvesting, suggesting that it plays a direct role in the development of obesity. The aim of this study was to investigate whether intestinal colonization by particular species/groups of the intestinal microbiota is related to body weight values in Estonian preschool children born in different years during the entire 1990s.

**Methods:**

Body weight, height, body mass index (BMI), and quantitative composition of cultivable gut microbiota (staphylococci, enterococci, streptococci, enterobacteria, lactobacilli, anaerobic gram-positive cocci, bifidobacteria, eubacteria, bacteroides, clostridia, and candida) were studied in 51 healthy 5-year-old children (40 were born between 1993 and 94 and 11 were born between 1996 and 97).

**Results:**

At the age of 5 years, median weight was 19.5 kg and median BMI was 15.3 kg/m^2^. Significantly higher BMI (*p*=0.006) was found in 5-year-old children born in late versus early 1990s during the development of socioeconomic situation of Estonia (2% rise in gross domestic product). The counts of the different gut bacteria did not show any association with weight and BMI in the 5-year-old children. However, the BMI values were in positive correlation with a relative share of anaerobic gram-positive bacteria, for example, bifidobacteria when adjusted for sex and year of birth (adj R^2^=0.459, *p*=0.026) and eubacteria (adj R^2^=0.484, *p*=0.014) in the community of cultured intestinal microbiota. The relative share of bacteroides showed a negative correlation with the childrens’ weight (adj R^2^=− 0.481, *p*=0.015).

**Conclusion:**

The body weight indices of preschool children of the general population are associated with the proportion of anaerobic intestinal microbiota and can be predicted by sex and particular socioeconomic situation from birth to 5 years of age.

The prevalence of metabolic diseases is increasing; in 2010, 43 million preschool children were estimated to be overweight or obese, and 92 million were at risk of being overweight. The worldwide prevalence of childhood overweight and obesity levels increased from 4.2% in 1990 to 6.7% in 2010 (1). Overweight and obesity levels remain major public health concerns because of serious consequences for health, including type 2 diabetes mellitus, cardiovascular diseases, pulmonary hypertension, obstructive sleep apnea, gastro-esophageal reflux disease, musculoskeletal disorders, a variety of cancers, and a number of psychological concerns (1, 2). The close association between gut microbiota composition has previously been associated with socioeconomic changes of lifestyle and prevalence of some diseases as allergy and metabolic syndrom (3–6). For instance, Estonian children born in the early 1990s had a significantly higher count of anaerobes such as *Bacteroidetes* and anaerobic gram-positive cocci than their 5-year-old counterparts born in the late 1990s, and they showed lower prevalence of allergic diseases (5, 7).

Recently, it has been shown that being overweight cannot be explained by genetic factors alone. According to the 16S RNA studies, a higher proportion of gram-positive *Firmicutes* phyla with low DNA G+C content (Bergey Manual) has been found in obese children, adults, and experimental animals in comparison to a low proportion of gram-negative *Bacteroidetes* phyla (8–11). On the contrary, Schwiertz (12) and Collado (13) with co-workers have indicated that overweight volunteers and pregnant women harbored significantly higher counts of the genus *Bacteroidetes* than lean volunteers and normal-weight pregnant women. However, these studies have applied different molecular methods, such as fluorescent *in situ* hybridization coupled with flow cytometry and quantitative real-time polymerase chain reactions. It is possible that the discrepancy is related to the inclusion of dead bacteria or such bacteria that do not express their metabolic potential due to different local epigenetic factors (14). Studies on cultivable microbial groups and their proportions in anthropometrically well-characterized populations would be helpful.

Kalliomäki et al. have pointed to the importance of early microbial colonization in the development of normal versus overweight children (15). Particularly during infancy, the *Firmicutes* comprising *Staphylococcus aureus* were prevalent in the intestinal tracts of 7-year-old children who were overweight. In comparison, in 7-year-olds with normal weight, the *Bifidobacterium* from phyla *Actinobacteria* were frequent gut colonizers during infancy (15). Similarly to children, obese adults have been shown to have a lower fecal concentration of *Bifidobacterium* and a higher concentration of *S. aureus* than their lean controls (12, 13).

However, there is not enough data showing that intestinal bacteria either of *Firmicutes* or other phyla are associated with higher or lower body weight in young children of general population without any known metabolic disorder. Preschool age seems to be a suitable term for investigation of the association between particular microbes and their metabolic impact on health, for at this time of life the gut microbiota composition is quite similar to that of adults due to the predominance of gram-negative *Bacteroidetes* over gram-positive *Bifidobacteria* and *Firmicutes*. This settled proportion of intestinal microbiota composition has been shown using different methods, such as viable counting procedures, measurements of 16S ribosomal RNA (rRNA) abundance, and the occurrence of specific signature fatty acids in whole community fatty acid methyl ester profiles (16, 17).

The present study aimed to investigate whether the intestinal colonization with particular species/groups of intestinal microbiota could be related to the body weight values in 5-year-old children of the general population. We further wanted to explore whether the lower versus higher socioeconomic development of Estonia during the years of growth of the preschool children in the 1990s versus the early 2000s could explain the association between microbiota and body weight.

## Material and methods

### Subjects

The study group comprised 51 Estonian children (23 male and 28 female): 40 (Group 1: 17 male and 23 female) of them born in 1993–94 and 11 (Group 2: 6 male and 5 female) born in 1996–97. All children were vaginally delivered without birth complications. The children were selected from a larger group in which the development of immune response to allergens and allergy was studied in relation to environmental factors (18, 19). The 5 years age in Group 1 was reached in the years 1997–1998, in Group 2 in the years 2001–2002. An acceleration in the socioeconomic development of Estonia was found in the 1990s – gross domestic product (GDP) per capita in Estonia increased from 1993 to 1997 by 38% and from 1998 to 2002 by 40% with a 2% difference.

The local ethics committee of the University of Tartu approved the study. The parents of all children signed their informed consent.

### Anthropometrical data

Each child was weighed in light clothing to the nearest 0.1 kg using a calibrated scale. Height was measured without shoes to the nearest 0.1 cm using a vertical ruler. Further, the body mass index (BMI) was calculated as weight (kg)/square meters of height.

Overweight was defined as BMI scores at or above the 85th percentile for age and gender, obesity at or above the 95th percentile. The overweight index was 18 and the obesity index was 20 for both boys and the girls at the age of 5 years.

### Bacteriological analyses

Stool samples (1–2 g) were collected and held in a domestic refrigerator at 4°C for no more than 2 hours before transportation to the laboratory, where they were frozen at −70°C until analyses. Weighed samples of feces were serially diluted (10^−2^–10^−9^) in pre-reduced phosphate buffer (pH 7.2) in the anaerobic glove box (Concept, UK) with a gas mixture of 5% CO_2_, 5% H_2_, and 90% N_2_. The quantitative analysis of the feces was performed by seeding serial dilutions on nine freshly prepared media (20).

Yeast extract agar was applied for total aerobes count; yeast extract agar with 6.5% of sodium chloride for staphylococci; Endo agar for enterobacteria; de Man-Rogosa-Sharpe agar (MRS; Oxoid, UK) for microaerobes, such as lactobacilli and streptococci; Wilkins-Chalgren agar (Oxoid, UK) for total anaerobes; Wilkins-Chalgren agar with vancomycin and nalidixic acid supplement (Oxoid, UK) for gram-negative bacteroides; Wilkins-Chalgren agar with colistin and nalidixic acid supplement for gram-positive bacteria, such as gram-positive anaerobic cocci, clostridia, bifidobacteria, and eubacteria; Cefoxitin-Cycloserine-Fructose agar (Oxoid, UK) with egg yolk and sodium taurocholate for *Clostridium difficile*. The Sabouraud dextrose agar with penicillin (50,000 U/L) and streptomycin (40,000 U/L) was applied for yeasts and fungi.

The colony counts of the different dilutions were recorded, and from the highest dilutions with growth all of the colonies with different morphologies were identified by standard methods, mostly on the genus and species level. After identification of microorganisms, which grew as single colonies in the dilutions, the quantitative composition of fecal microbiota was determined. The number of various species or genus was given as colony-forming unit per gram of feces (CFU/g) expressed in log_10._ The detection level was ≥3log CFU/g. For each child, the counts of different bacterial groups were calculated and summarized to obtain the total count of cultivable intestinal bacteria. The relative share (%) of each bacterial group was calculated from total counts.

### Statistics

The statistical analysis was performed using the SIGMASTAT 2.0 (Jandel Scientific Corporation, USA) and SPSS 11.0 (SPSS Inc., Chicago, IL, USA) statistical software package.

According to data, the descriptive statistics, the χ^2^-test or Fisher exact test, and Student's *t*-test or the Mann–Whitney rank sum test were applied to compare the prevalence and composition of gut bacteria in children. The linear correlation test and the multiple linear regression models were used to test the association between the microbiological data and body weight or BMI. *P*-values less than 0.05 were considered statistically significant.

## Results

At the age of 5 years, the children's median of body weight was 19.5 kg (range 14.5–33 kg) and BMI 15.3 kg/m^2^ (range 12.7–23.1 kg/m^2^). Two children were diagnosed as overweight, and one child was obese. BMI of children born in 1993–94 was lower than BMI of children born in 1996–97 (*p*=0.006; Table 1).

**Table 1:** Clinical data (birth-weight, breast-feeding, body weight, height, and BMI of 5-year-old children)

	Children born in different years		
		
Clinical data	Group 1: born in 1993–1994 (*n*=40; mean±std dev)	Group 2: born in 1996–1997 (*n*=11; mean±std dev)	*P*
Birth-weight (g)	3,573±587	3,527±457	0.813
Breast-fed (months)	6±2	7±5	0.731
Body weight (kg)	19.2±2.3	21. 9±4.6	0.064
Body height (cm)	112.7±4.1	114.1±4.7	0.329
BMI (kg/m^2^)	15.1±1.3	16.7±2.5	0.006

The prevalence and counts of gut bacteria in children born 1993–94 versus 1996–97 were not statistically different (Table 2). However, in children born in 1993–94, the relative share of anaerobic gram-positive cocci (*p*=0.002) was higher, and the relative share of coagulase negative staphylococci (*p*=0.043) was lower than those in their counterparts born later.

**Table 2:** The prevalence (%), the counts (log10; CFU/g), and relative share (%) of gut microorganisms in children born in different years

	Children born in different years						
				
	Group 1: born in 1993–1994 (*n*=40)		Group 2: born in 1996–1997 (*n*=11)		All children (*n*=51)		
			
Microorganisms	Prevalence number (%)	Counts (log CFU/g); median range	Prevalence number (%)	Counts (log CFU/g) median (range	Prevalence number (%)	Counts (log CFU/g) median range	Relative share in total count mean (%) SD
CONS	23 (58)	6.3 (3.3–10.2)	5 (45)	6.0 (5.4–9.3)	28 (54)	6.3 (3.3–10.2)	2.6±6.3
*S. aureus*	16 (40)	4.9 (3.3–8.3)	2 (18)	5.8 (4.3–7.3)	18 (35)	4.9 (3.3–8.3)	0.08±0.2
Enterococci	27 (68)	6.6 (3.6–10.4)	9 (82)	6.3 (3.3–9.6)	36 (71)	6.6 (3.3–10.4)	3.1±6.0
Enterobacteria	38 (95)	7 (4.3–9.3)	10 (91)	8.2 (5.4–9.6)	48 (94)	7.3 (4.3–9.6)	2.2±4.8
Streptococci	16 (40)	8.6 (4.2–10.1)	8 (73)	6.8 (4.3–10.6)	24 (47)	7.6 (4.2–10.6)	7.1±10.9
Lactobacilli	19 (48)	5.9 (4–10.4)	7 (64)	4.6 (3.3–9.3)	26 (50)	5.5 (3.3–10.4)	3.9±8.8
Anaerobic gram-positive cocci	34 (85)	9.6 (6.3–10.8)	10 (91)	9.3 (7.3–10.3)	44 (86)	9.6 (6.3–10.6)	27.3±22.4
Bifidobacteria	10 (25)	9.3 (6.3–10.6)	3 (27)	8.6 (7.6–9.2)	13 (25)	9.2 (6.3–10.6)	24.7±20.6
Eubacteria	12 (30)	9.3 (7.3–10.3)	6 (55)	9.8 (7.8–10.3)	18 (35)	8.8 (6.8–11.1)	17.6±22.1
Bacteroides	32 (80)	8.6 (3.3–11.1)	11 (100)	10.3 (7.8–11)	43 (84)	9.9 (3.3–11.1)	44.1±29.8
Clostridia	21 (53)	8.0 (3.8–10.3)	8 (72)	8.4 (4.1–10.3)	29 (57)	8.0 (3.8–10.3)	8.7±14.2
Candida	11 (28)	5.3 (3.3–6.6)	1 (9)	3.6	12 (24)	5.2 (3.3–6.6)	0.009±0.02

CONS, coagulase negative staphylococci.

**Fig. 1:**
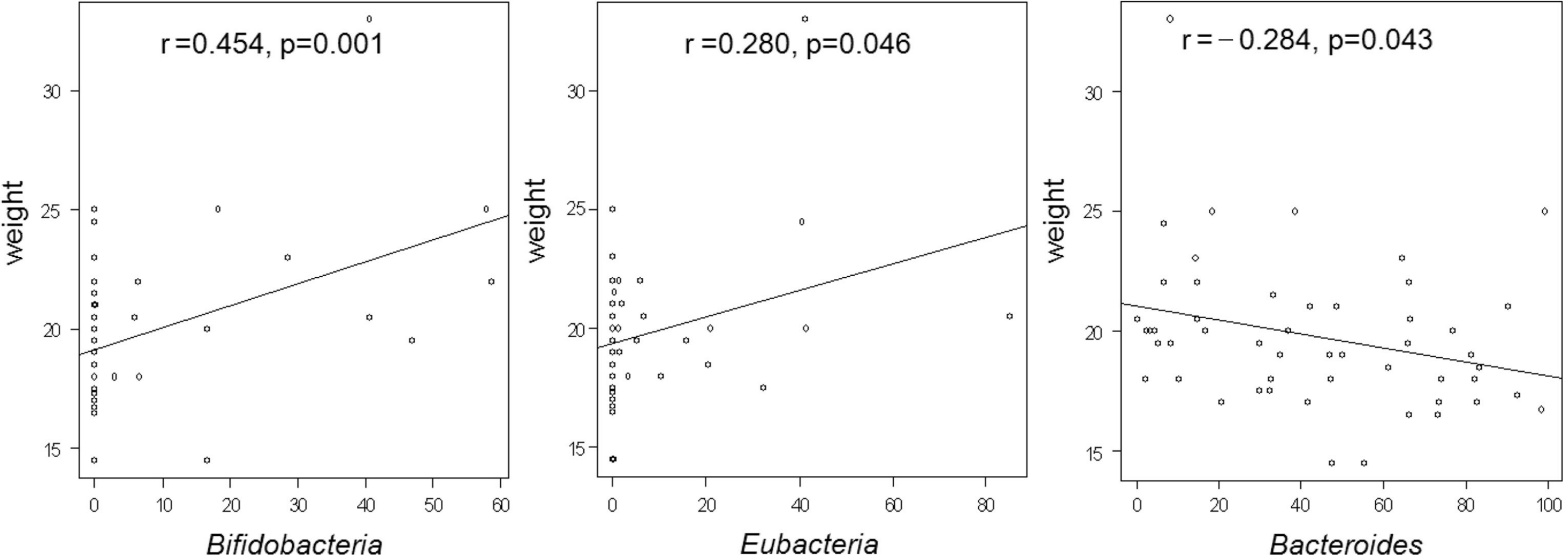
Correlation between body weight (kg) of 5-year-old children and relative share (%) of eubacteria, bifidobacteria, and bacteroides.

The counts of different gut bacteria did not express any association between weight and BMI in the children. At the same time, the anthropometrical indices of 5-year-old children showed close association with the relative share of some groups of anaerobic bacteria in the whole community of estimated microbiota: bifidobacteria with body weight (r=0.454, *p*=0.001) and BMI (r=0.352, *p*=0.001), and eubacteria with body weight (r=0.280, *p*=0.046) and BMI (r=0.328, *p*=0.019) (Table 3; Fig. 1). In contrast, the relative share of bacteroides expressed a negative correlation only with the childrens’ weight (r=−0.284; *p*=0.043; Fig. 1).

**Table 3:** Linear and multiple linear regression analysis between body weight, BMI, and different gut microbes of the children

Variable 1	Variable 2	Linear correlation (*r*- and *p*-values)	Multiple linear regression analysis adjusted for sex and year of birth (Adj R^2^, *p*-values)
Weight (kg)	Bifidobacteria (log_10_; CFU/g)	r=0.454, *p*=0.001	Adj R^2^=0.51, *p*=0.006
	Eubacteria (log_10_; CFU/g)	r=0.280, *p*=0.046	Adj R^2^=0.448, *p*=0.033
	Bacteroides (log_10_; CFU/g)	r=−0.284, *p*=0.043	Adj R^2^=−0.481, *p*=0.015
BMI (kg/m^2^)	Bifidobacteria (log_10_; CFU/g)	r=0.352, *p*=0.001	Adj R^2^=0.459, *p*=0.026
	Eubacteria (log_10_; CFU/g)	r=0.328, *p*=0.019	Adj R^2^=0.484, *p*=0.014

The indices are adjusted for sex and year of birth.

After adjustment for sex and year of birth, both weight and BMI were positively correlated with the relative share of bifidobacteria (body weight r=0.51, *p*=0.006; BMI r=0.459, *p*=0.026; Table 3) and eubacteria (body weight r=0.448, *p*=0.033; BMI r=0.484, *p*=0.014; Table 3). Weight was negatively correlated to bacteroides (r=-0.481, *p*=0.015; Table 3).

## Discussion

We have assessed that in 5-year-old children the microbial balance between different groups of anaerobic intestinal microbiota is associated with the values of body weight. Our study group comprised Estonian preschool children with mainly normal weight, only three of the children having a BMI over the 85th percentile. However, the BMI was generally higher in children born at the end of 1990s. The definition of obesity in children involves BMI greater than the 85th (commonly used to define overweight) or the 95th (commonly used to define obesity) percentile for age-matched and sex-matched control subjects (21).

In the 5-year-old children under examination, we found that the higher values of body weight and BMI were related to a higher proportion of bifidobacteria and eubacteria. Moreover, besides the aforementioned groups of microbes, body weight was also associated with a low proportion of bacteroides in total numbers of cultivable intestinal microbiota. To the best of our knowledge, this is the first study by bacterial method that associates the composition of gut microbiota with body weight and BMI on the basis of sex and year of birth in preschool children of the general population.

The application of a relative share of particular groups of bacteria for characterization of intestinal microbiota helped us to overcome some methodological shortages of cultivation of bacteria on special media (5, 20, 22). Our finding supports the data that abundance of phylum *Bacteroidetes* is connected with lower host weight and correlated negatively with BMI (8–11, 23). Besides the confirmation of the involvement of the *Firmicutes* group, such as *Eubacterium* in the higher values of body weight, we have assessed the divergent role of *Bifidobacteria* in 5-year-old children, similar to adults versus infants (12, 15). Similar to our study, Turnbaugh and co-workers revealed a higher proportion of phyla *Actinobacteria*, including bifidobacteria, among obese subjects (24), and contrary to our study, Kalliomäki and co-workers have found that early colonization with bifidobacteria at infancy might lower the risk of obesity later in life (15). The association between increased relative share and being overweight may be connected with the colonization of the gut with different species of *Bifidobacterium*.

The question remains as to how the high proportions of *Bifidobacterium* and low proportions of *Bacteroidete* of the gut microbiota can cause increased BMIs in the host. Some differences are apparent in the carbohydrate metabolism between bifidobacteria and bacteroides of particular species, which might predispose or protect against overweight and obesity. The total short-chain fatty acid (SCFA) concentration in fecal samples of obese subjects was more than 20% higher than in lean volunteers (12). When carbohydrate intake was lowered, the butyrate-producing *Firmicutes* group decreased and the propionate proportion and propionate-producing *Bacteroidetes* counts went higher in overweight than in lean volunteers (12). Propionate may inhibit lipid synthesis from acetate as shown in rat hepatocytes (25). Besides, SCFA receptor deficient mice are leaner than their wild-type counterparts, further implicating SCFAs as signaling molecules in the development of obesity (26). In general, the capacity to ferment carbohydrates to SCFA varies greatly among bacterial species and also among *Lactobacillus* sp. strains (27). Thus, it has been proposed that the actual composition of the intestinal microbial flora in a given person may be an individual contributor to host energy metabolism.

Bäckhed et al. have speculated that a change in microbial ecology is the result of a Western diet, and/or that differences in intestinal microbial ecology between individuals may function as ‘environmental’ factors affecting energy storage and obesity (28). In our study, BMI increased in Estonian children in accordance with the accelerating socioeconomic development of Estonia over the 1990s. The 2% increase of GDP per capita during different periods of growth of the two studied groups of children (1993–1997 vs. 1998–2002) up to reaching 5 years of age has been documented. This coincides well with the 1.3% increase in the number of overweight children in the 10–13 age group between the years 2007 and 2008 (data of Estonian Health Insurance Fund, http://www.haigekassa.ee/eng/ehif).

An energy-rich diet of Western type could have led to an increase in BMI of the preschool children under scrutiny in our study. However, the particular differences in the microbiota composition, that is, the higher relative share of staphylococci and lower proportion of anaerobic cocci in children born in the late 1990s could not be attributed to the BMI values in the children of our study. On the contrary, in the whole group of 5-year-old children of the general population predicted by sex and year of birth, there was significant association with the composition of some groups of anaerobic intestinal microbiota.

Karlsson with co-workers indicated that the prevalence of gram-negative *Enterobacteriaceae* were significantly higher in the obese/overweight compared to normal weight preschool children (29). The Western diet-induced dysbiosis of gut and low diversity of microbiota can lead to many inflammatory diseases (6). However, in our study, the relative share of enterobacteria was not bound with higher body weight and BMI as much as our study group comprised only three children who were overweight and/or obese.

Thus, we showed that BMI increased with the advancing socioeconomic development of Estonia from the 1990s to 2000s. In conclusion, the body weight indices of preschool children of the general population are associated with the proportion of anaerobic intestinal microbiota and can be predicted by sex and the particular socioeconomic situation from birth to 5 years of age.
